# Defense against oxidative stress in *Caenorhabditis elegans* by dark tea

**DOI:** 10.3389/fvets.2023.1342747

**Published:** 2024-01-05

**Authors:** Jianxiu Wang, Kaiheng Zhang, Yaya Zhang, Shumin Ge, Shuhua Zhang

**Affiliations:** School of Life Science and Technology, Changchun University of Science and Technology, Changchun, China

**Keywords:** dark tea, oxidative stress, longevity, mRNA sequencing analysis, *Caenorhabditis elegans*

## Abstract

Dark tea, rich in nutricines including tea polyphenols and free amino acids, is a kind of post-fermented tea. The potential application of nutricines against oxidative damage and senescence, which drives animal health maintenance and disease prevention, has attracted considerable interest. In this study, the effect of dark tea and its effects on longevity and defense against oxidative stress was investigated in the *Caenorhabditis elegans* (*C. elegans*) model. Under normal conditions, dark tea extended the lifespan without significant impairment of propagation. It also improved the motility, alleviated the fat accumulation and apoptosis. Additionally, orally administered dark tea could significantly decrease the level of reactive oxygen species (ROS) and resulted in a superior lifespan in H_2_O_2_-induced oxidative stressed *C. elegans*. In antioxidant assays *in vitro*, dark tea was found to be rich in strong hydroxyl, DPPH and ABTS+ free radical scavenging capacity. Interestingly, mRNA sequence analyses further revealed that dark tea may catalyze intracellular relevant oxidative substrates and synthesize antioxidants through synthetic and metabolic pathways. These results suggest that dark tea is worth further exploration as a potential dietary supplement for the maintenance of animal health and the prevention of related diseases.

## 1 Introduction

The health of farm animals is critical to production and profitability. Alterations in energy metabolism can adversely affect their health. Elevated metabolic demands can lead to a significantly increased oxygen demand which can result in an upsurge in reactive oxygen species (ROS) (1). This, in turn, disrupts the equilibrium between the production and elimination of free radicals, contributing to a heightened inflammatory response (2). Disruptions in the redox balance have been associated with common diseases such as enteritis and mastitis in pigs and cattle, and recurrent airway obstruction in horses (3). However, the currently used chemically synthesized antioxidants have been under suspicion for their association with organic damage (4, 5). The efficient production of foods at a low cost is of great importance for good health. Therefore, researchers have developed a keen interest on the potential application of natural nutrients against oxidative damage and for longevity.

Tea is a commonly consumed functional beverage. It has high levels of safety and significant therapeutic effects, together with low toxicity and minimal side effects. According to the different production methods, tea can be classified into white tea, green tea, yellow tea, oolong tea, black tea, and dark tea. The quality characteristics of tea are associated with a combination of various active substances, including polyphenolic compounds for astringency, free amino acids for the sense of newness, and volatile substances for aromas (6, 7). Tea has a variety of health properties, such as antioxidant, anti-inflammatory, immunomodulatory, anti-cancer, cardio-protective, anti-diabetic, weight loss and hepatoprotective effects (8). The available data suggest that green tea polyphenol supplementation at postpartum improved the milk yield and health status in cows with hyperketonemia during early lactation (9). Green tea extracts could improve intestinal microflora balance, contributing to the prevention of digestive and respiratory organ diseases in calves (10). The catechins components in green tea have been found to relieve oxidative stress and fatty liver disease in dairy cows during the periparturient phase and transition period (11, 12). Hence, tea can be used as a functional substance in livestock feed.

Dark tea is a tree belonging to the *Camellia sinensis* family, distinguished by the oily black or black-brown color of its leaves (13). The process of production of dark tea involves microorganisms; hence, dark tea is the only post-fermented tea among the six major tea types (14). Compared to the widely studied green, black, and oolong teas, dark tea is often overlooked as a unique post-fermented tea. Dark tea contains phytochemicals and macronutrients, also believed to be beneficial to animals (15). As a natural and harmless nutraceutical, it can be used as a nutritional supplement, food additive and medicinal ingredient (16, 17).

*Caenorhabditis elegans* (*C. elegans*) is the first multicellular organism to have its genome sequenced fully. The conservation of illnesses-related pathways between *C. elegans* and higher organisms, along with the advantages of its short life cycle, simple culture and high reproduction capacity (18, 19), has made *C. elegans* a favorable *in vivo* non-rodent model organism for mechanistic explanations and high-throughput screening of drug candidates, being screened for a range of oxidative stress, toxicity and related conditions or diseases (20).

Therefore, in this study, three types of Chinese dark tea, all of which are commonly available in the market and have high sales volumes, were selected as raw materials, namely Brick tea, Pu'er tea, and Liubao tea, to investigate the effects of dark tea on the lifespan, propagation, motility, fat deposition, apoptosis, and resistance to oxidative stress in *C. elegans*. Additionally, the differences in the expression of genes were explored by the mRNA sequence analyses.

## 2 Materials and methods

### 2.1 Preparation of dark tea extract

Brick tea (Anhua, China), Pu'er tea (Xishuangbanna, China), or Liubao tea (Wuzhou, China), were mixed with water in a 1:15 ratio of tea to water, and heated in a water bath at 85°C for 1 h, cooled and filtered, and then subjected to rotary evaporation at 58°C for 1 h, respectively (21).

### 2.2 Active ingredients in dark tea extract

The total polyphenol contents of dark tea were determined through the iron tartrate colorimetric method (22). Free amino acids were detected through ninhydrin colorimetry (23). The active ingredients in dark tea extract are listed in Supplementary Table 1.

### 2.3 Free radical scavenging ability of dark tea

Determination of the free radical scavenging ability of Brick, Pu'er, and Liubao teas at concentrations of 200, 400, 600 and 800 μg/mL, respectively.

#### 2.3.1 Hydroxyl radical scavenging ability of dark tea

A sample solution (0.5 mL) of the dark tea extracts was added to 0.5 mL of salicylic acid ethanol solution (9 mmol/L; LABGO, China), 0.5 mL of H_2_O_2_ (9 mmol/L; Guangfu, China) solution, and 0.5 mL of FeSO_4_ (9 mmol/L; BEIJINGSHIJI, China) solution. The mixtures were allowed to react for 30 min in a 37°C water bath, and the absorbance of the resulting solution was measured at 510 nm, and the values were assessed against a blank (24). The hydroxyl radical scavenging ability was calculated by the following formula:


$$\mathrm{Hydroxyl}\;\mathrm{radical}\;\mathrm{scavenging}\;\mathrm{activity}\;\left(\%\right)=\left(1-\frac{A1-A2}{A0}\right)\times 100\%$$

where A1 is the absorbance of the reaction solution with the sample, A2 is the absorbance of the solution without salicylic acid and A0 is the control group where the sample was replaced with distilled water.

#### 2.3.2 DPPH free radical scavenging ability of dark tea

Dark tea extracts (2 mL) were added to 2 mL of DPPH (Aladdn, China) ethanol solution (5 mg/mL). The mixtures were allowed to react for 30 min in a dark place, and the absorbance of the resulting solution was measured at 517 nm, and the values were calculated against a blank (25). The DPPH radical scavenging ability was calculated by the following formula:


$$\mathrm{DPPH}\;\mathrm{radical}\;\mathrm{scavenging}\;\mathrm{activity}\;\left(\%\right)=\left(1-\frac{A1-A2}{A0}\right)\times 100\%$$

where A1 is the absorbance of the reaction solution with the sample, A2 is the absorbance of anhydrous ethanol in place of DPPH and A0 is the absorbance of anhydrous ethanol in place of sample.

#### 2.3.3 ABTS+ free radical scavenging ability of dark tea

Dark tea extracts (3 mL) were added to 1 mL of 2,2′-azino-bis(3-ethylbenzthiazoline-6-sulphonate) (ABTS; Rhawn, Chian) solution. The mixtures were allowed to react for 6 min in a 30°C water bath, and the absorbance of the resulting solution was measured at 734 nm, and the values were assessed against a blank (26). The ABTS+ radical scavenging ability was calculated by the following formula:


$$\mathrm{ABTS}+\mathrm{radical}\;\mathrm{scavenging}\;\mathrm{activity}\;\left(\%\right)=\left(1-\frac{A1}{A2}\right)\times 100\%$$

where A1 is the absorbance of the reaction solution with the sample and A2 is the control group where the sample was replaced with distilled water.

### 2.4 *Caenorhabditis elegans* strains and maintenance

In this research, the wild-type N2 strain of *C. elegans* was used. All *C. elegans* strains were grown in the nematode growth medium (NGM) at 20°C, and all *C. elegans* feed on *Escherichia coli OP50*. All *C. elegans* strains and the *Escherichia coli OP50* were purchased from the Caenorhabditis Genetics Centre, University of Minnesota (Minneapolis, MN, USA). *C. elegans* was transferred by cutting a small portion of the nematode-containing NGM using a sterile scalpel, placing it upside down in OP50-coated NGM, and incubating it at 20°C to allow *C. elegans* to crawl onto the new NGM. At the time of synchronization during the spawning period, *C. elegans* were washed down with M9 buffer, the lysate was added, and the precipitate was taken by centrifugation at low speed.

### 2.5 Lifespan assay

The synchronized N2 *C. elegans* were placed on NGM containing Brick tea extract (600, 700, 800 μg/mL), Pu’er tea extract (600, 700, 800 μg/mL), and Liubao tea extract (600, 700, 800 μg/mL) for incubation, which was set up as the experimental group, and the one with no dark tea extract as the control group. To block *C. elegans* reproduction, 5-Fluorouracil (FUDR; Rhawn, China) was added. For each strain, at least 100 egg-synchronized *C. elegans* were grown at 20°C and transferred daily to a fresh plate (27). The number of *C. elegans* surviving, dying and lost was recorded every day until all *C. elegans* were dead.

### 2.6 Lifespan assay in the oxidative damage state

The synchronized N2 *C. elegans* were placed on NGM containing 600 μg/mL Brick tea extract, 700 μg/mL Pu’er tea extract, and 700 μg/mL Liubao tea extract for cultivation. They were allowed to grow until reaching the L4 stage after which cultivation was continued for an additional 4 days and compared with the control group. Oxidative damage to *C. elegans* was induced by adding 200 μL of H_2_O_2_ (0.5 mmol/L, Rhawn, China) solution to a 96-well plate (28). Then 15 *C. elegans* were placed into each well, and the number of *C. elegans* that died was observed at hourly intervals until all of the *C. elegans* were dead, and the survival rate was estimated.

### 2.7 Progeny assay

The method was the same as mentioned in Section 2.6. During the reproductive period (approximately days 1–6), *C. elegans* were transferred every day to new NGM plates and allowed to deposit progeny. Record the number of eggs laid per day (29).

### 2.8 Motility assay

The same method as mentioned in Section 2.6 was followed. FUDR (Rhawn, China) was added to block *C. elegans* reproduction and then incubated at 20°C until days 2, 4, 8, 12, and 14. *C. elegans* were then picked and placed onto agar spacers coated with OP50. The frequency of head bobbing in 1 min and the number of body bending within 20 s were observed using a body microscope (30).

### 2.9 Fat deposition assay

For this, the protocol mentioned in Section 2.6 was followed. Synchronized *C. elegans* were grown for 4 days after which they were washed with M9 buffer, and the precipitate was collected after low-speed centrifugation. Subsequently, 100 μL of 1% paraformaldehyde (Skyho, China) was added, and the sample was kept at 4°C for 15 min before being snap-frozen and stored at −80°C. After 1 h, the samples were removed, thawed, centrifuged, and rinsed with M9 buffer. Next, a mixture of equal volumes of 2% Triton X-100 (Applygen, China) and 1% Oil Red O (Solarbio, China) was prepared, and 100 μL of this mixture was added for precipitation. The mixture was then incubated in a 37°C temperature-controlled shaker for 30 min (31). Finally, *C. elegans* were picked and placed on an agar spacer using a picker, and images were captured under a fluorescence microscope.

### 2.10 Reactive oxygen species (ROS) accumulation assay

The DCFH-DA method (32) was used to detect the level of ROS *in vivo*. ROS levels in *C. elegans* were measured under both normal conditions and following induction of oxidative damage by H_2_O_2_. Same method as Section 2.6. Subsequently, the *C. elegans* were exposed to H_2_O_2_ (0.5 mmol/L) for 2 h at 20°C. The OP50 around *C. elegans* was washed using M9 buffer; from this, 100 μL of precipitate was taken and 1 μL of 10 mM H2DCF-DA (Chemstan, China) was added, and placed in a constant temperature shaker at 37°C in dark for 30 min. *C. elegans* was then transferred to a 2% agar spacer, and observed and photographed using a fluorescence microscope at the excitation wavelength of 485 nm and the emission wavelength of 528 nm.

### 2.11 Apoptosis assay

Apoptosis was experimentally detected *in vivo* using acridine orange staining (33), following the same method mentioned in Section 2.6. After three rinses *C. elegans* with M9 buffer, the supernatant was aspirated, and 100 μL of acridine orange (25 μg/mL, Klamar, China) staining solution was added and placed on a constant temperature shaker at 37°C, in the dark for 2 h. Subsequently, *C. elegans* were placed in blank NGM for 10 min to allow recovery from the staining. Finally, they were transferred to 2% agar pads and observed under a fluorescence microscope at an excitation wavelength of 488 nm and an emission wavelength of 515 nm.

### 2.12 Transcriptomics analysis

After 4 days of feeding on dark tea, *C. elegans* was rinsed, placed in microfuge tubes, and left to settle, after which the precipitate was taken. RNA-seq was performed on a sequencing platform at Beijing BMK Biotechnology Co., Ltd. (Beijing, China), to obtain insights into aggregate gene transcription in subject *C. elegans* cells.

### 2.13 Statistical analysis

Statistical analysis was performed using GraphPad Prism 8, and expressed as mean ± standard deviation (SD). One-way ANOVA was used for multiple group comparisons. Values with *p* < 0.05 were considered statistically significant. The intensity of quantified fluorograms was analyzed using ImageJ software. Each experiment was repeated three times.

## 3 Results

### 3.1 Dark tea extends the lifespan of *Caenorhabditis elegans*

**Figure 1:**
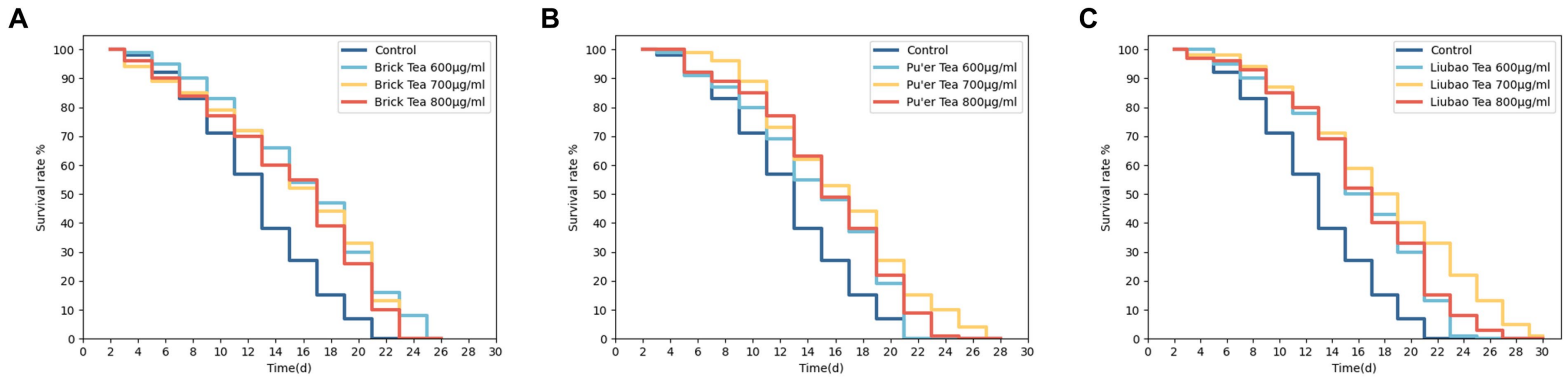
Effects of various concentrations of dark tea (**A**, Brick Tea; **B**, Pu’er Tea; **C**, Liubao Tea) on the lifespan in *C. elegans*.

The *C. elegans* were successively treated with Brick, Pu'er, and Liubao teas under normal culture conditions. As shown in Figure 1 and Table 1, all these treatments affected the normal *C. elegans* lifespan. The mean lifespan of control *C. elegans* was 14 days. Treatment with the different teas was found to prolong the lifespan of *C. elegans*, resulting in increased average lifespan of 21.4, 14.3, and 14.3% after treatment with Brick tea at concentrations of 600, 700, and 800 μg/mL, respectively, 7.1, 21.4, and 14.3% after exposure to the same concentrations of Pu’er tea, and 21.4, 28.4, and 21.4% for Liubao tea, respectively, all relative to the control group. In addition, 600 μg/mL Brick tea, 700 μg/mL Pu'er tea, and 700 μg/mL Liubao tea had the most significant effect on the increase in average and maximum lifespan (Table 1). Therefore, the above-mentioned concentrations were selected for subsequent experiments.

**Table 1:** Mean and maximum lifespan in *C. elegans* (Mean ± SD).

Group	Mean lifespan (d)	Maximum lifespan (d)	Lifespan improvement rate%^a^
Control	13.76 ± 1.67	20 ± 2.05	
600 μg/ml Brick Tea	17.2 ± 1.70	22 ± 2.48	20
700 μg/ml Brick Tea	16.24 ± 1.77	22 ± 1.7	15.3
800 μg/ml Brick Tea	16.14 ± 1.60	22 ± 2.05	14.7
600 μg/ml Pu’er Tea	15.7 ± 1.85	20 ± 1.63	12.4
700 μg/ml Pu’er Tea	17.44 ± 1.05	26 ± 2.05	21.1
800 μg/ml Pu’er Tea	16.5 ± 1.62	24 ± 1.63	16.6
600 μg/ml Liubao Tea	17.08 ± 1.37	24 ± 2.24	19.4
700 μg/ml Liubao Tea	18.7 ± 1.51	30 ± 1.63	26.4
800 μg/ml Liubao Tea	17.42 ± 1.57	26 ± 1.64	21

a Lifespan improvement rate%: Maximum life increase/Maximum lifespan of the control.

### 3.2 Dark tea enhances the oxidative stress resistance in *Caenorhabditis elegans*

#### 3.2.1 Dark tea extends the lifespan of *Caenorhabditis elegans* under oxidative damage conditions

**Figure 2:**
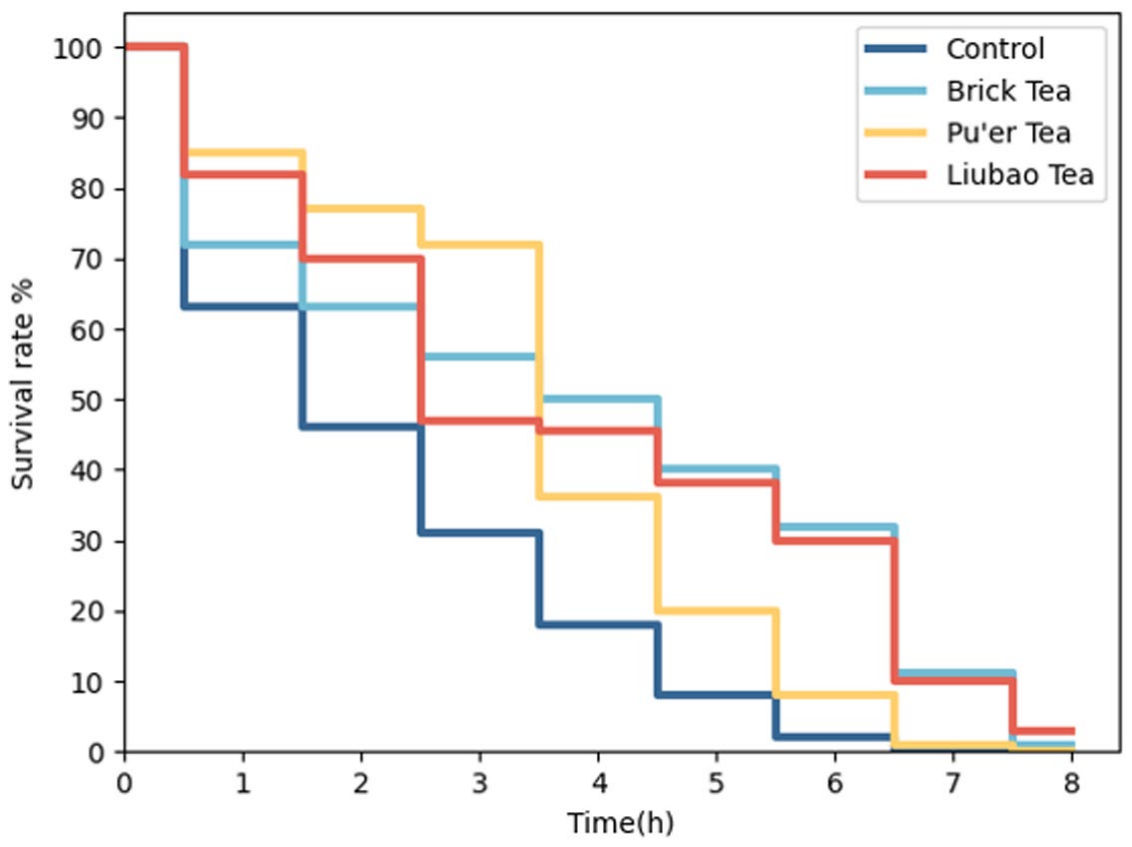
Effects of dark tea on the lifespan in *C. elegans* under oxidative damage conditions.

Lifespan involves the process of gradual aging, and the oxidative damage pathway accelerates aging (34). Under oxidative stress, the lifespan of dark tea-treated *C. elegans* increased significantly, especially in those treated with Brick tea or Liubao tea (Figure 2).

#### 3.2.2 Dark tea decreases the ROS level of *Caenorhabditis elegans*

**Figure 3:**
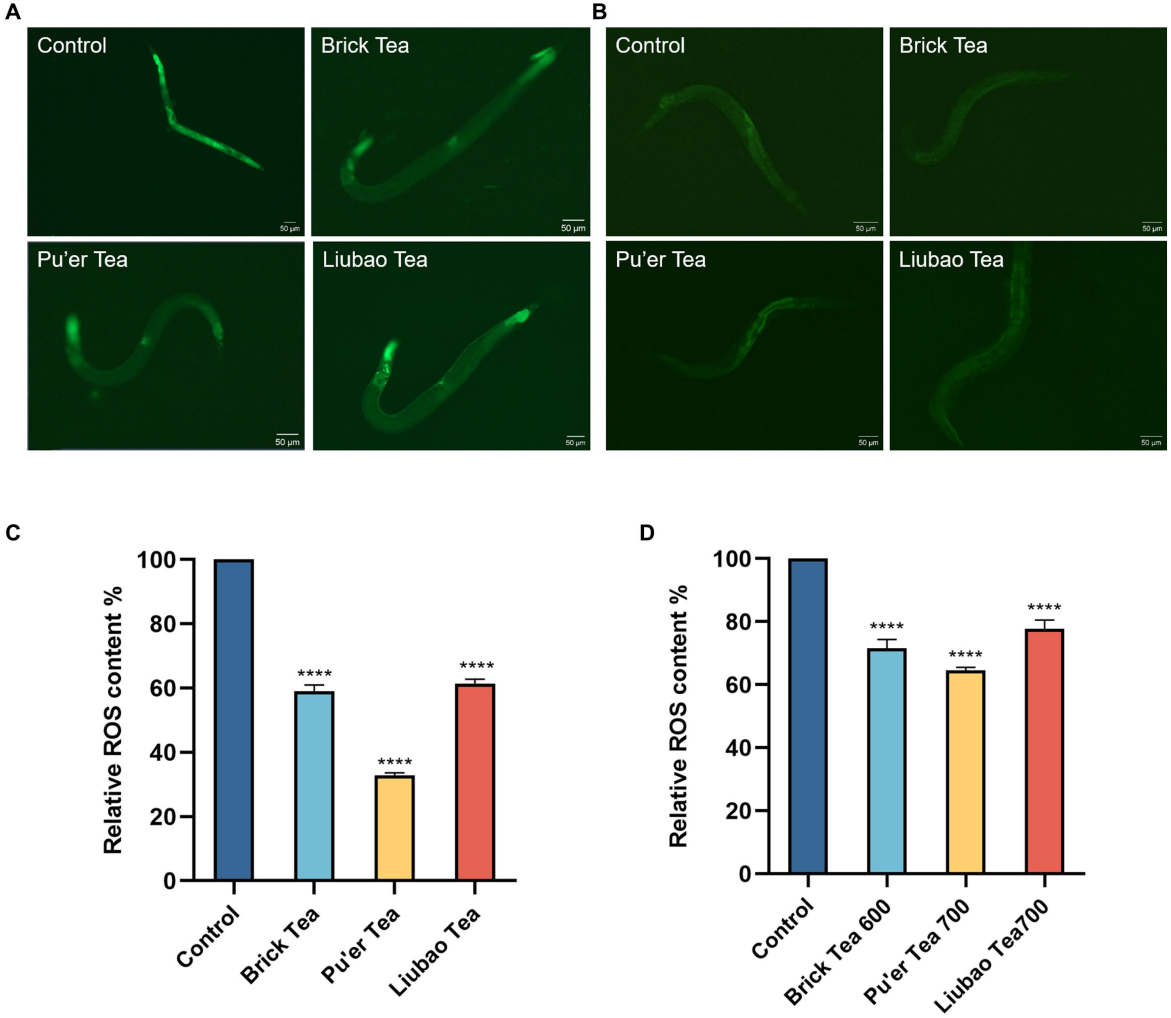
Effects of dark tea on the ROS levels in *C. elegans*. **(A)** ROS fluorescence plot in the normal state. **(B)** ROS fluorescence plot in the oxidative damage state. **(C)** Quantitative analysis plot of ROS fluorescence intensity in the normal state. **(D)** Quantitative analysis plot of ROS fluorescence intensity in the oxidative damage state. **** indicates *p* < 0.0001.

Under both normal conditions and oxidative stress, the control group had the highest ROS level and this effect was minimized by dark tea treatments (Figure 3). The fluorescence intensity decreased by 41, 68, and 37% after treatment with Brick, Pu’er, and Liubao teas, respectively (Figure 3C), without H_2_O_2_ treatment, and decreased by 28.4, 35.4, and 22.2%, respectively (Figure 3D), in the presence of H_2_O_2_ compared with the control group. These results demonstrated that dark tea markedly reduced the levels of ROS in *C. elegans*, indicating that dark tea has strong antioxidant activity *in vivo*.

#### 3.2.3 Dark tea scavenges hydroxyl, DPPH, and ABTS+ free radical *in vitro*

**Figure 4:**
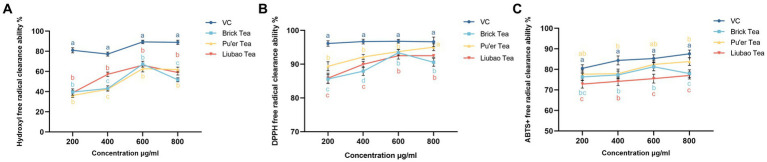
Effects of dark tea on the free radicals scavenging capacity. **(A)** -OH. **(B)** DPPH. **(C)** ABTS+.

To further assess the effects of dark tea on rendering stress resistance in *C. elegans*, the free radicals scavenging capacity of dark tea was investigated. As shown in Figure 4, the scavenging capacity of 600 μg/mL of Brick tea for hydroxyl, DPPH, and ABTS+ radicals was maximized. The scavenging ability of Pu’er tea and Liubao tea for DPPH, and ABTS+ radicals increased with increasing concentration; however, the scavenging ability for hydroxyl radicals reached a maximum at 600 μg/mL. These results suggest the strong free radicals scavenging capacity of dark tea.

### 3.3 Dark tea exerts no toxicity on the fertility of *Caenorhabditis elegans*

**Figure 5:**
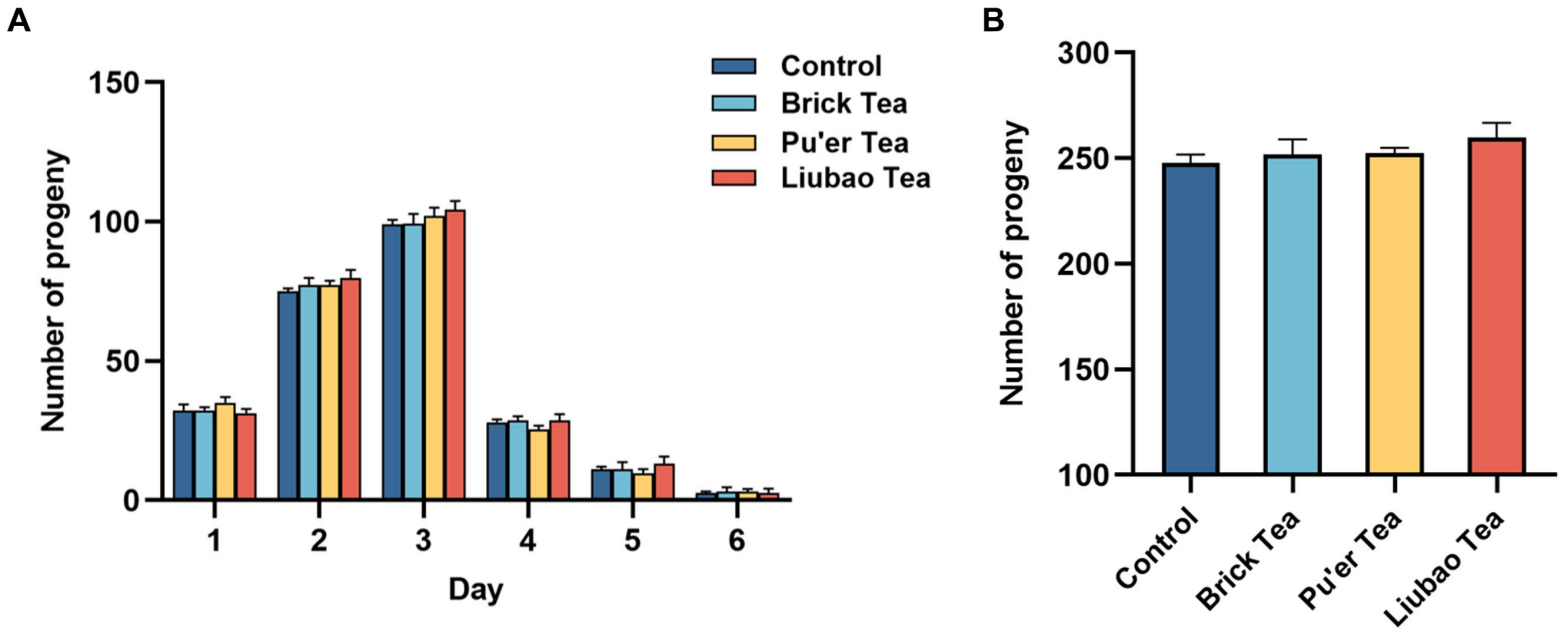
Effects of dark tea on the reproductive capacity in *C. elegans*. **(A)** The number of offspring daily in each groups in the pawning period. **(B)** The total of number of offspring in each group in the pawning period.

The spawning period of *C. elegans* was about 6–7 days, and mainly concentrated in the first 3 days (Figure 5A). There was no significant difference between the experimental groups in terms of the total number of offspring (Figure 5B). The results indicated that the reproductive capacity of *C. elegans* is not impaired by dark tea and provided evidence for the safety of dark tea.

### 3.4 Dark tea promotes the motility of *Caenorhabditis elegans*

**Figure 6:**
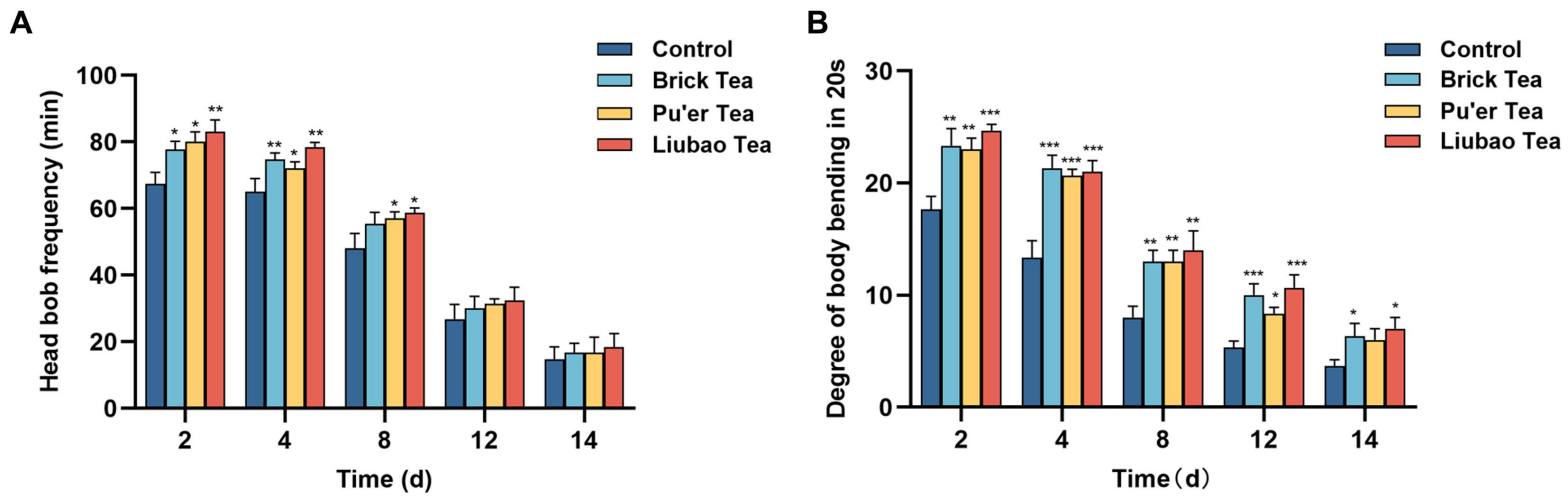
Effects of dark tea on the motility in *C. elegans*. **(A)** Frequency of head bobbing in 1 min. **(B)** Degree of body bending in 20 s. * indicates *p* < 0.05, ** indicates *p* < 0.01, *** indicates *p* < 0.001.

With aging, muscle function gradually declines, which in turn slows down mobility. The effect of dark tea on motility was recorded on days 2, 4, 8, 12 and 14 post-dosing, and the results are presented in Figure 6. On day 12, the motility of *C. elegans* decreased substantially. The dark tea significantly affected the frequency of head bobbing in *C. elegans* in the pre-preliminary stage, among these, Liubao tea had the most significant effect (Figure 6A). Dark tea substantially enhanced the body bending frequency of *C. elegans*, whereas the effect of Pu’er tea on *C. elegans* in the later stages of the process was not significant (Figure 6B). Thus, dark tea can alleviate the aging-related decrease in the energy transportation capacity of *C. elegans*.

### 3.5 Dark tea alleviates the accumulation of fat of *Caenorhabditis elegans*

**Figure 7:**
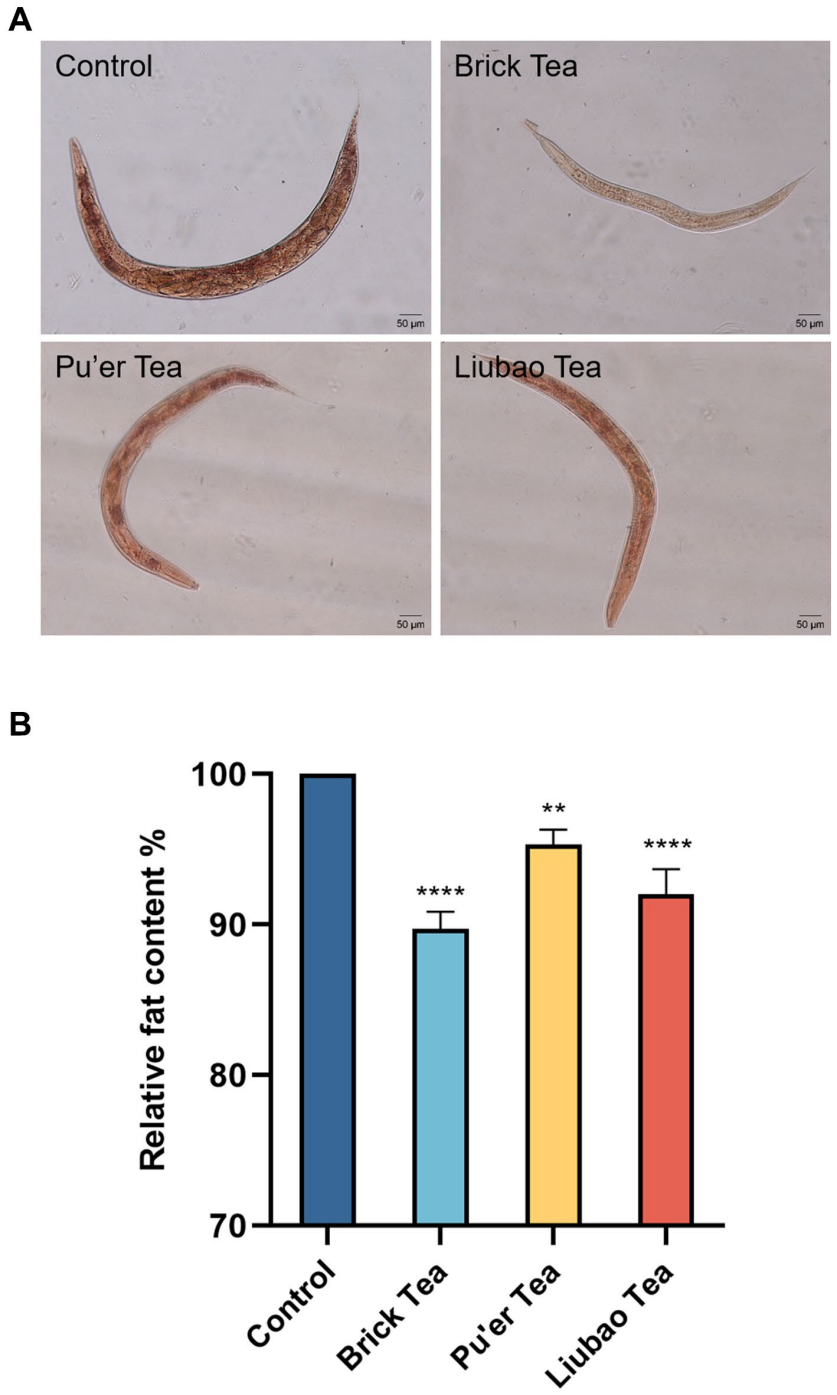
Effects of dark tea on the fat content in *C. elegans*. **(A)** Plot of fat staining. **(B)** Plot of quantitative analysis of staining intensity. ** indicates *p* < 0.01, **** indicates *p* < 0.0001.

Fat is the root cause of inflammation due to aging (35). Staining with Oil Red O (Solarbio, China) showed a significantly lower number of fat cells in the experimental group than those in the control group (Figure 7A), and *C. elegans* fed Brick tea had the lowest body fat content. Further analysis revealed that the body fat content of *C. elegans* fed with Brick tea, Pu’er tea, or Liubao tea was reduced by 9, 4, and 8%, respectively (Figure 7B). These results suggest that dark tea significantly ameliorated age-associated physiological characteristics by reducing fat deposition in *C. elegans* by reducing fat accumulation.

### 3.6 Dark tea reduced the apoptosis of *Caenorhabditis elegans*

**Figure 8:**
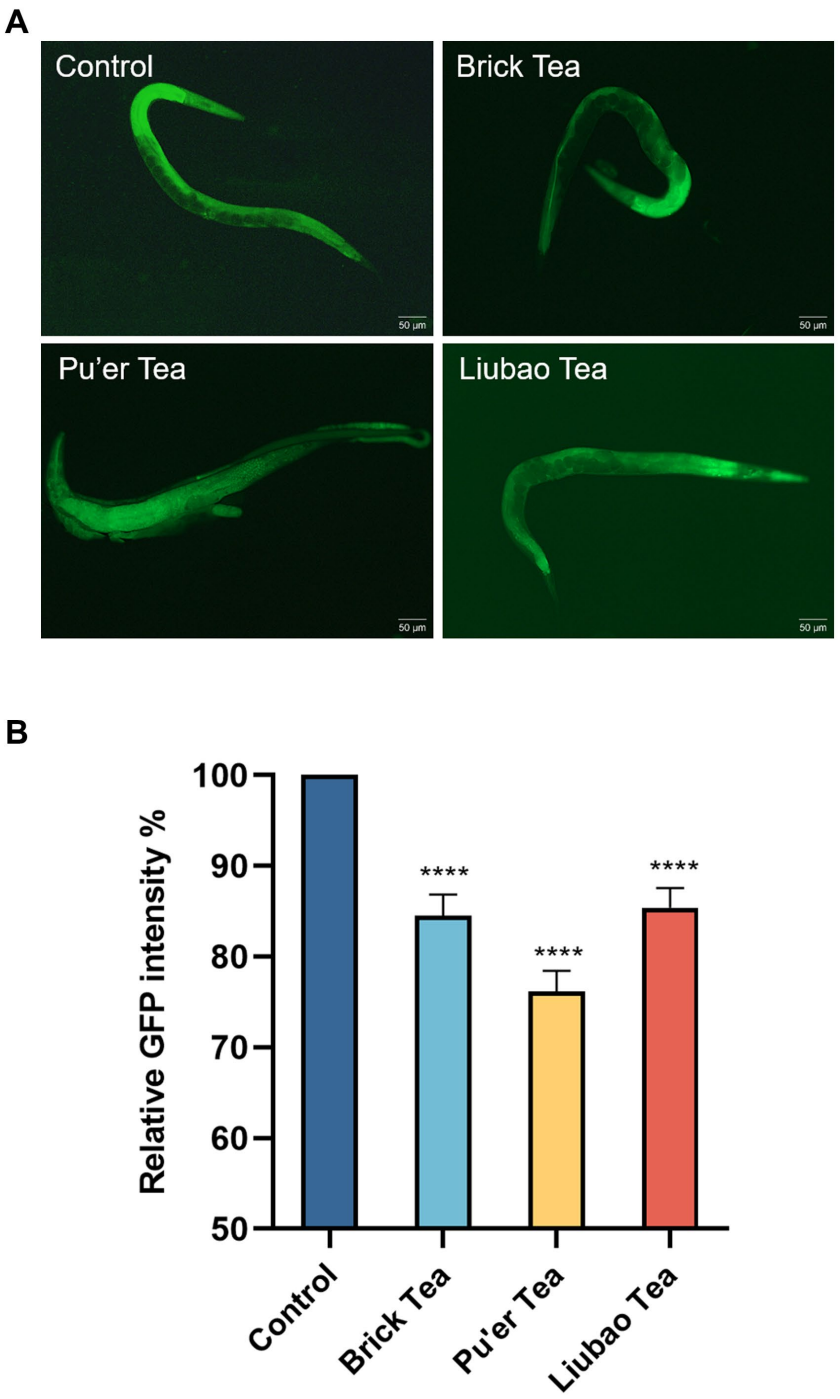
Effects of dark tea on the apoptosis in *C. elegans*. **(A)** Apoptosis fluorescence graph. **(B)** Quantitative analysis graph of apoptosis fluorescence intensity. **** indicates *p* < 0.0001.

*Caenorhabditis elegans* treated with Brick tea, Pu'er tea, and Liubao tea had significantly lower apoptosis than the control group (Figure 8A). Quantitative analysis of the fluorescence intensity in *C. elegans* fed Brick tea, Pu’er tea, and Liubao tea decreased by 15, 24, and 14%, respectively (Figure 8B). The above results show that dark tea could achieve anti-aging effects by inhibiting the degree of apoptosis.

### 3.7 Transcriptomics analysis

#### 3.7.1 Gene expression differences

**Figure 9:**
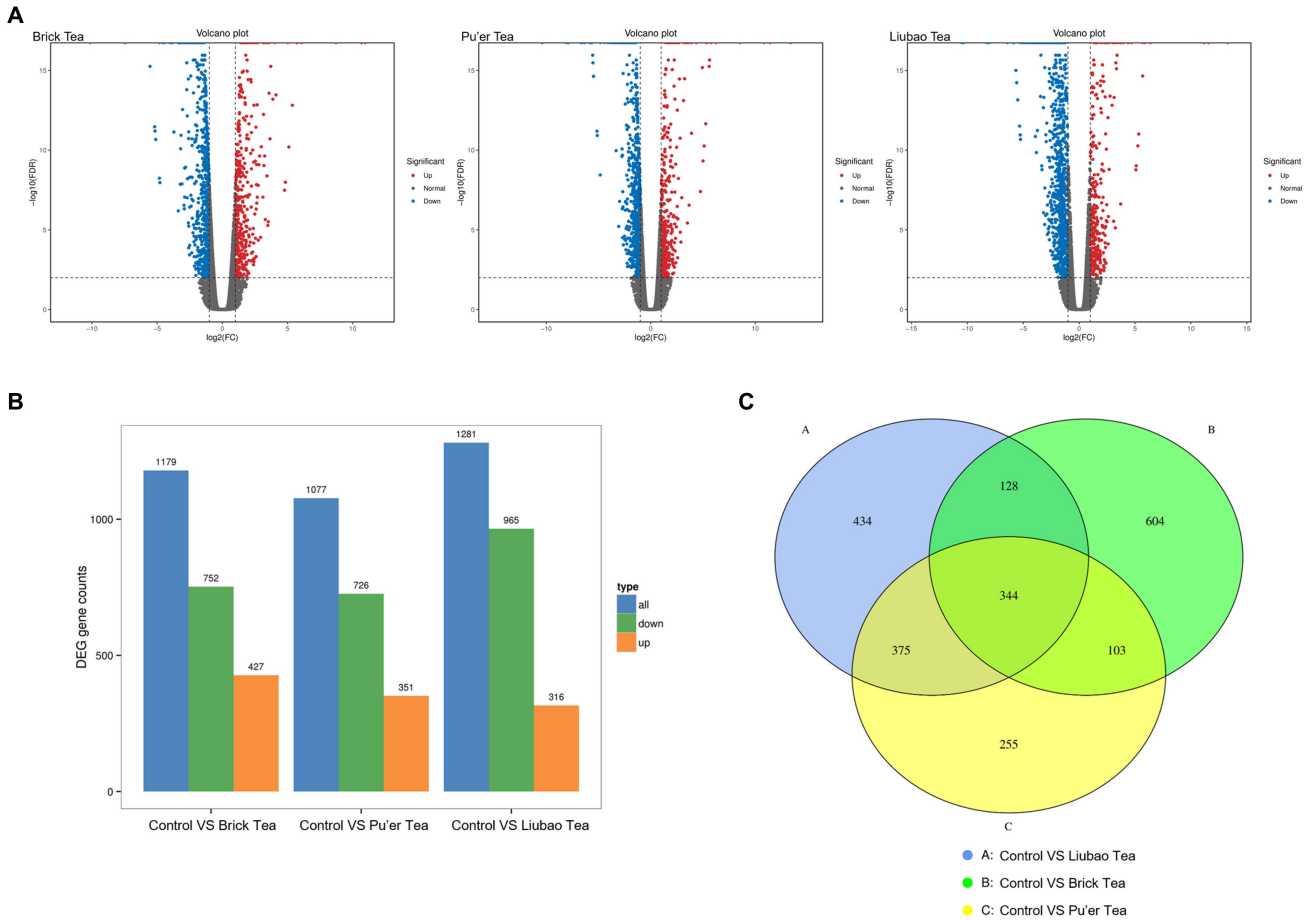
Distribution of genes identified in dark tea. **(A)** Volcano plot of differentially expressed genes. The horizontal coordinate indicates the logarithmic value of the fold difference in expression of a gene in the two samples and the vertical coordinate indicates the negative logarithmic value of the statistical significance of the change in gene expression. Red dots indicate up-regulation and blue dots indicate down-regulation. **(B)** Up-regulated and down-regulated genes. **(C)** Venn diagram of the set of differential genes (fold change≥2).

Genetic data of dark tea-treated three groups are shown in Figure 9. The volcano plot (red, up-regulated; blue, down-regulated) revealed that the number of down-regulated or up-regulated genes in the Brick tea, Pu’er tea, and Liubao tea groups overlapped, while each group also exhibited unique genes (Figure 9A). Interestingly, the number of down-regulated genes was the highest in the Liubao tea group, while the number of up-regulated genes was the most in the Brick tea group was the most (Figure 9B). Compared to the control group, the same 344 genes were identified in the Brick tea, Pu’er tea, and Liubao tea groups (Figure 9C).

#### 3.7.2 GO enrichment of differentially quantified genes

**Figure 10:**
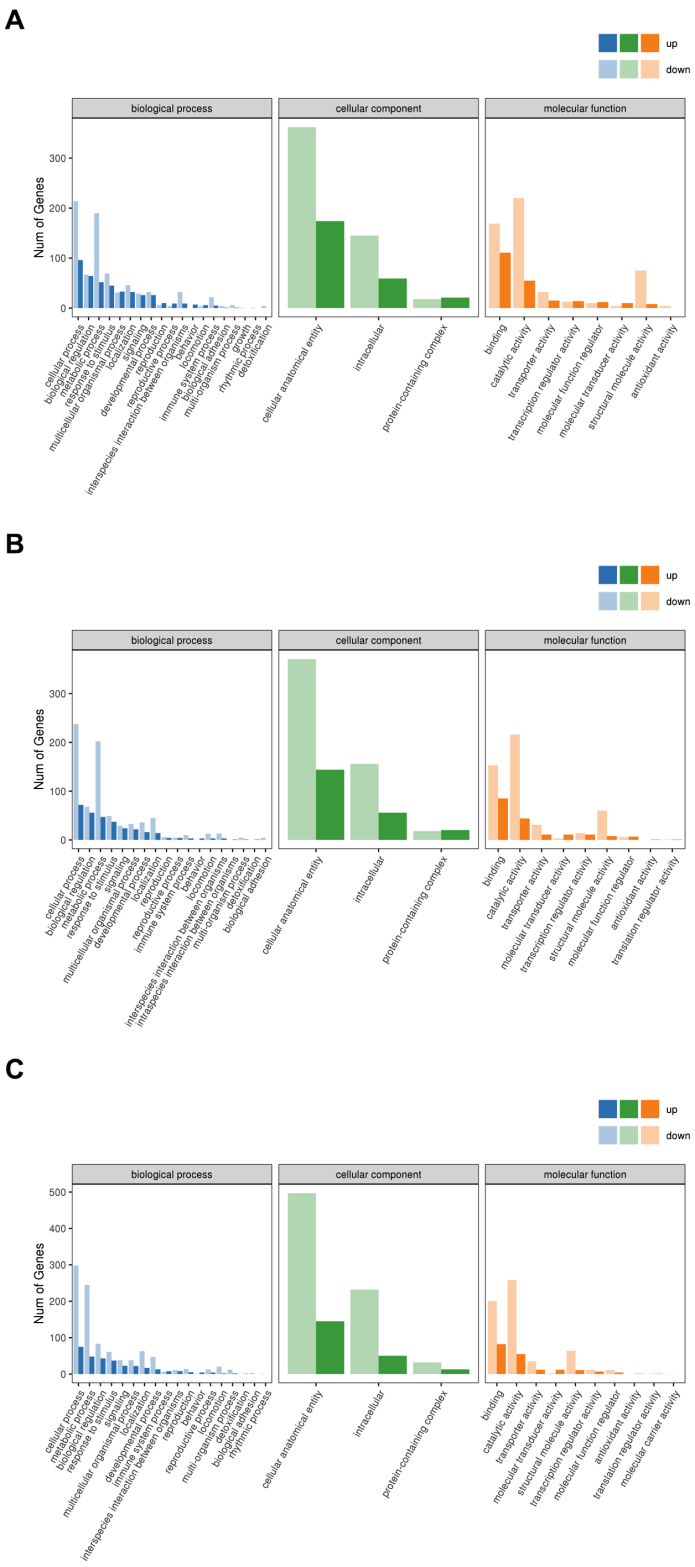
GO annotation categorization statistical chart. **(A)** Brick Tea. **(B)** Pu’er Tea **(C)** Liubao Tea. The horizontal coordinate is the GO classification, the vertical coordinate is the number of genes, and different colors represent the different primary classifications to which they belong.

The gene products were analyzed for GO enrichment in terms of biological process (BP), cellular component (CC), and molecular function (MF), as shown in Figure 10. A few of the same up-regulated and down-regulated genes with functional annotations were identified in the Brick tea, Pu’er tea, and Liubao tea groups, which included cellular process and metabolic process in BP, cellular anatomical entity in CC, and catalytic activity and binding in MF. The alterations in the above-mentioned biological functions have all been shown to affect lifespan (36). Thus, dark tea mainly affects endocrine system-related pathways, especially catalysis-related bio-metabolism within cells.

#### 3.7.3 Specific regulation pathways of dark tea

**Figure 11:**
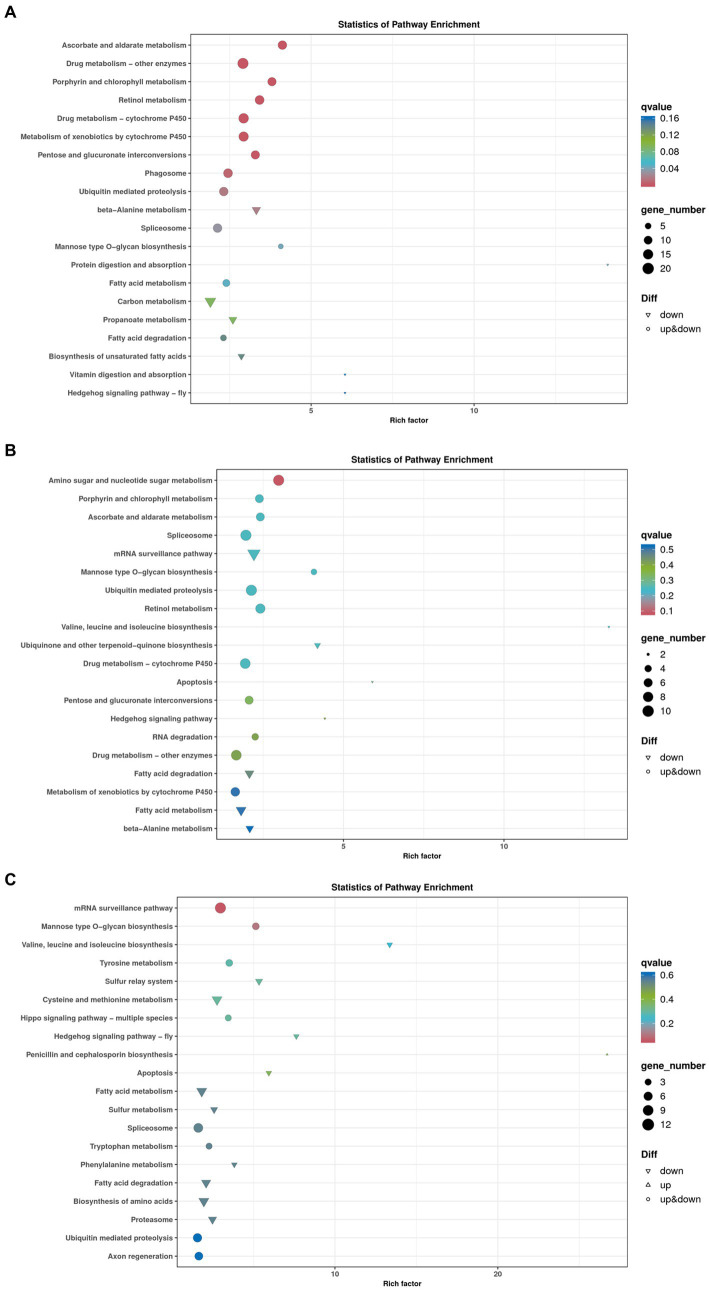
Bubble chart of genes enriched in KEGG pathways. **(A)** Brick Tea. **(B)** Pu’er Tea **(C)** Liubao Tea. The bubble size represents the number of genes in the enriched pathway terms, and the bubble color represents the *p* value.

To further explore the regulation pathways of dark tea, a KEGG pathway enrichment analysis was conducted (Figure 11). In the Brick tea, Pu’er tea, and Liubao tea groups, the ubiquitin mediated proteolysis, spliceosome and mannose type O-glycan biosynthesis pathways were significantly up-regulated, while fatty acid metabolism and fatty acid degradation pathways were significantly down-regulated. Among the annotated pathways, a majority were closely linked to glucose and lipid metabolism. These pathways suggested the metabolism and biosynthesis are significantly involved in the redox reaction, which were important to improve antioxidant defense system.

A total of 2, 1, and 2 genes, respectively, were significantly up-regulated (*p* < 0.05) in the ubiquitin mediated proteolysis, mannose type O-glycan biosynthesis, and fatty acid metabolism and degradation pathways, respectively (Table 2). Specifically, the expression of PRKN and SKP1 in the ubiquitin mediated proteolysis was significantly up-regulated (*p* < 0.05). After dark tea treatment, the expression of K12H6.3 in the mannose type O-glycan biosynthesis was significantly up-regulated (*p* < 0.05); in addition, the expression of ACADM, CPT1 in the fatty acid metabolism and degradation pathways was significantly up-regulated (*p* < 0.05).

**Table 2:** Genes after dark tea intervention in the ubiquitin mediated proteolysis, mannose type O-glycan biosynthesis, fatty acid metabolism and fatty acid degradation pathways in *C. elegans*.

Pathways	No	Gene name	Regulated	Log2FC^a^
Ubiquitin mediated proteolysis	1	PRKN	Up	1.43
	2	SKP1	Up	2.63
Mannose type O-glycan biosynthesis	1	K12H6.3	Up	3.32
Fatty acid metabolism and degradation	1	ACADM	Down	−1.17
	2	CPT1	Down	−1.48

a Log2FC indicates differences in gene expression in the comparison of two samples.

## 4 Discussion

As the only post-fermented tea, many biochemical reactions of the fermentation process in the production of dark tea result in differences that set dark tea and other five types of tea. Twenty-two macronutrient chemical composition indicators in 65 Chinese dark teas have been reported, with the richer ones being polyphenols and free amino acids (9). In the present study, we first determined the content of the main two nutrients in the three represented dark teas. The contents of tea polyphenols and L-theanine in dark tea were maintained at around 10 and 1.5% (Supplementary Table 1).

Free radical production is essential in normal metabolism, and excessive free radical formation may lead to oxidative stress and other related diseases (37). Xu et al. (38) found that tea polyphenols increased SOD, CAT, T-AOC and GSH-Px contents along with a reduction in MAD and ROS in crows. In this study, we found that orally supplied dark tea could significantly decrease the ROS levels and exhibited a superior lifespan in H_2_O_2_-induced oxidative stress *C. elegans*. Similarly, we observed an improvement in-OH, DPPH, and ABTS+ free radicals scavenging capacity in antioxidant assays *in vitro*, indicating the scavenging ability of dark tea.

The relationship between the activity of antioxidants and longevity promotion has long been noticed. Although many chemically synthesized antioxidants are available, long-term use may have potential side effects (2, 3). We found that dark tea extended the lifespan and motility without significantly impairing propagation in *C. elegans*. Obesity due to excessive fat accumulation is associated with accelerated onset of diseases occurring in old age, while fat ablation increases life span (39). Apoptosis is also considered an effective immune defense mechanism when the body is subjected to various harmful stimuli (40, 41). Our results indicated that the lipid oxidation products and apoptosis in *C. elegans* could be reduced after dark tea treatment. Therefore, several conclusions and speculations can be drawn, which suggest that the longevity extension of dark tea might be attributed to the inhibition of lipid oxidation and the extent of generation of apoptosis-related products to protect the health of the body. Noticeably, no significant toxic effects of dark tea were observed in our study.

GO analysis allows for a standardized description of the gene products in terms of the biological pathways involved and cellular localization (42). To elucidate the roles of the identified differentially expressed genes, GO analysis revealed significant enrichment of BP, CC, and MF, namely, cellular process, metabolic process, catalytic activity, and binding, which play independent roles in antioxidant and anti-aging traits. According to KEGG enrichment analysis the number of genes annotated to a metabolic pathway in a differentially expressed gene is significantly greater than the proportion of background genes among all such genes (43). Among the annotated pathways, most are related to glucose and lipid metabolism. Four common enriched signaling pathways identified in three dark tea groups included mannose type O-glycan biosynthesis, fatty acid metabolism, fatty acid degradation, ubiquitin mediated proteolysis, and spliceosome. Further comparison of the differentially expressed genes with the functional annotations of the genes in the database, revealed that most of the genes with more significant changes in gene up-regulation and down-regulation because of dark tea treatment were regulated by SKP1, K12H6.3, ACADM, and CPT1. SKP1 was up-regulated in the ubiquitin mediated proteolysis pathway, which connects cell cycle regulators to the ubiquitin proteolysis machinery to thus improve cellular growth (44). K12H6.3 was found to be involved in the mannose type O-glycan biosynthesis pathway; the gene encodes the relevant core α1,3-fucosyltransferase, whose expression is promoted during development (45). ACADM and CPT1 are key genes catalyzing mitochondrial fatty acid metabolism and degradation. They catalyze the rate-limiting step of the conversion of acyl-coenzyme. As into acyl-carnitines, which can cross membranes to enter the mitochondria, and elevate triglyceride, phospholipid, and droplet levels of cellular lipids to regulate genetic, epigenetic, physiological, and nutritional modulators (46).

Overall, it may be safely concluded that dark tea can independently reduce oxidative stress, thus preventing oxidative injury. As a natural and non-toxic antioxidant, dark tea can be used as a nutritional supplement for livestock and as a functional component of livestock feed, thus acting as a defense against various diseases caused by oxidative damage. It thus has great promise for animal health and disease prevention. Further exploration of dark tea as a potential dietary supplement for animal health maintenance and disease prevention is worth exploring.

## 5 Conclusion

In conclusion, the safety of dark tea was demonstrated in *C. elegans* and dark tea was found to mitigate oxidative stress-induced damage by promoting the clearance of free radicals. Additionally, it was observed that dark tea modulated metabolic processes, including carbohydrates, lipids, and proteins, catalyzing the synthesis of antioxidants from specific oxidative substrates within the cell.

## Data availability statement

The datasets presented in this study can be found in online repositories. The names of the repository/repositories and accession number(s) can be found at: https://figshare.com/, 10.6084/m9.figshare.24609774.

## Ethics statement

The animal study was approved by Animal Care and Use Committee, Changchun University of Science and Technology. The study was conducted in accordance with the local legislation and institutional requirements.

## Author contributions

JW: Writing – original draft, Writing – review & editing. KZ: Writing – original draft. YZ: Writing – original draft. SG: Writing – review & editing. SZ: Writing – review & editing.
