# Supplementary material for: Defense against oxidative stress in Caenorhabditis elegans by dark tea
Source: Front Vet Sci. 2024 Jan 5;10:1342747. doi: 10.3389/fvets.2023.1342747 (PMC10796627; doi:10.3389/fvets.2023.1342747)
Supplement: Supplementary file 1 [file Table_1.DOCX]

Supplementary Table 1 The active ingredient composition of Brick tea, Pu;er tea, and Liubao tea

| Items | Dark Tea | | |
| --- | --- | --- | --- |
|  | Brick Tea | Pu’er Tea | Liubao Tea |
| Tea Polyphenols | 6.91±0.52 | 12.54±0.58 | 10.79±0.69 |
| L-Theanine | 1.38±0.27 | 2.15±0.36 | 1.60±0.26 |

^a^Tea Polyphenols% = f $\frac{\rho nV}{106m}$ × 100% ρ-Mass concentration of gallic acid on the standard curve, n-Quality of tea, m-Dilution factor, V-Total volume of the sample. L-Theanine% = $\frac{c}{m\times1000}$ × 100% c- Mass number of amino acids on the standard curve, m- Determination of the sample solution equivalent to the mass of the sample.
